# Elucidating the ensemble of functionally-relevant transitions in protein systems with a robotics-inspired method

**DOI:** 10.1186/1472-6807-13-S1-S8

**Published:** 2013-11-08

**Authors:** Kevin Molloy, Amarda Shehu

**Affiliations:** 1 Department of Computer Science, George Mason University, 4400 University Dr., Fairfax, VA, 22030, USA; 2 Department of Bioengineering, George Mason University, 4400 University Dr., Fairfax, VA, 22030, USA; 3 School of Systems Biology, George Mason University, 10900 University Blvd., Manassas, VA, 20110, USA

## Abstract

**Background:**

Many proteins tune their biological function by transitioning between different functional states, effectively acting as dynamic molecular machines. Detailed structural characterization of transition trajectories is central to understanding the relationship between protein dynamics and function. Computational approaches that build on the Molecular Dynamics framework are in principle able to model transition trajectories at great detail but also at considerable computational cost. Methods that delay consideration of dynamics and focus instead on elucidating energetically-credible conformational paths connecting two functionally-relevant structures provide a complementary approach. Effective sampling-based path planning methods originating in robotics have been recently proposed to produce conformational paths. These methods largely model short peptides or address large proteins by simplifying conformational space.

**Methods:**

We propose a robotics-inspired method that connects two given structures of a protein by sampling conformational paths. The method focuses on small- to medium-size proteins, efficiently modeling structural deformations through the use of the molecular fragment replacement technique. In particular, the method grows a tree in conformational space rooted at the start structure, steering the tree to a goal region defined around the goal structure. We investigate various bias schemes over a progress coordinate for balance between coverage of conformational space and progress towards the goal. A geometric projection layer promotes path diversity. A reactive temperature scheme allows sampling of rare paths that cross energy barriers.

**Results and conclusions:**

Experiments are conducted on small- to medium-size proteins of length up to 214 amino acids and with multiple known functionally-relevant states, some of which are more than 13Å apart of each-other. Analysis reveals that the method effectively obtains conformational paths connecting structural states that are significantly different. A detailed analysis on the depth and breadth of the tree suggests that a soft global bias over the progress coordinate enhances sampling and results in higher path diversity. The explicit geometric projection layer that biases the exploration away from over-sampled regions further increases coverage, often improving proximity to the goal by forcing the exploration to find new paths. The reactive temperature scheme is shown effective in increasing path diversity, particularly in difficult structural transitions with known high-energy barriers.

## Background

Many proteins undergo large conformational changes that allow them to tune their biological function by transitioning between different functional states, effectively acting as dynamic molecular machines [1]. In most cases, either no structural information exists on the intermediate conformations in a transition trajectory, or this information is rather limited. One reason for the scarcity of structural information is the inability of experimental techniques to structurally track a transition. Probing the transition at the sub-nanometer scale, as required to elucidate structures along the transition, is in principle possible with spectroscopic techniques, such as FRET or NMR. However, doing so in practice is difficult, as the actual time spent during a transition event can be short compared to the long time a protein can send in a stable or meta-stable state. Exceptions exist, and some multi-functional proteins have been caught in the act. On many well-studied systems, such as Calmodulin and Adenylate Kinase, which are also subjects of our investigation in this paper, not only have the diverse functional structures been mapped, but some intermediate structures have also been elucidated. There are now many crystal structures deposited for the stable and/or intermediate states of these two systems in the Protein Data Bank (PDB) [2].

Since it is generally difficult for experimental techniques to provide detailed information regarding a transition trajectory and its intermediate conformations, computational techniques provide an alternative approach to computing transition trajectories and so attaining insight into the dynamic nature of proteins [3]. Doing so with reasonable computational resources remains challenging [4], as transition trajectories may span multiple length and time scales. In terms of length scale, some transition trajectories have been found to connect structural states more than 100Å apart of each-other in conformational space. For comparison, this is up to 2 orders of magnitude larger than the typical interatomic distance of 2Å. In terms of time scale, some transitions can demand *μ*s-ms time scales, which are 6*-*12 orders of magnitude larger than the typical fs-ps atomic oscillations.

Given the length and time scales possibly spanned by a transition, one can then draw analogies between computing transition trajectories and folding, where the start structure is a representative of an unfolded rather than some stable or meta-stable state. Analogies with folding make exploration methods that build on the classic Molecular Dynamics (MD) and Monte Carlo [5] frameworks typically used to elucidate folding and unfolding processes in proteins valuable computational tools to compute general transition trajectories.

By taking into account dynamics, MD-based methods provide detailed information into the time scales associated with conformational changes. However, given the length and time scales involved in non-trivial transitions, these methods can demand great computational resources and even be prohibitively expensive. Typically, MD-based methods are more appropriate to simulate a protein in its native or equilibrium environment and can do so at atomistic detail [6-8]. However, navigating the energy surface of a protein in search of rare transition trajectories with equilibrium MD-based methods remains very costly. A simulation may spend a long time in a local minimum corresponding to a stable or semi-stable state and only rarely undergo a conformational change allowing it to cross an energy barrier and transition to another state. Long simulation times may be needed to capture the rare event in a transition trajectory, which makes equilibrium MD-based methods as inadequate as the NMR and FRET experimental techniques in this setting.

Many adaptations are pursued to lower the computational demands of MD-based methods. Essentially, MD-based methods for elucidating transitions incorporate some suitable bias at the expense of obtaining possibly different transition trajectories. Methods include umbrella sampling, importance sampling, targeted, biased, or steered MD, local flattening of energy surface, conformational flooding, activation relaxation, replica exchange, swarm methods, and others [9-20]. To address computational demands, coarse graining and techniques based on normal mode analysis and elastic network modeling are typically used [21-31]. Some methods first generate a conformational path (morphing is an example of a technique that allows to do so trivially) and then spend resources on improving the path's energy profile. Methods in this category include the nudged elastic band, zero-temperature string, and finite-temperature string methods [32-38]. While the incorporation of a suitable bias towards the goal structure forces the simulation to reduce dwell time in a given stable or meta-stable state, the bias possibly sacrifices a more expansive view of possibly different transition trajectories to the goal structure. This is typically addressed by repeating the simulation to obtain many transition trajectories, which taken together can cover the transition ensemble if there are no correlations between them.

Since simulation of dynamics is the limiting factor in dynamics-based methods, efficiency concerns can be addressed by foregoing or at least delaying dynamics until credible conformational paths have been obtained. A different class of methods focus not on producing transition trajectories but rather computing a sequence of conformations (a conformational path) with a credible energy profile. The working assumption is that credible conformational paths can be locally deformed with techniques that consider dynamics to obtain transition trajectories. Most notably, methods in this category adapt sampling-based search algorithms developed for the robot motion-planning problem. The motion planning problem in robotics bears strong analogies to the problem of computing conformations along a structural transition. The objective in robot motion planning is to obtain paths that allow a robot to transition from a start configuration to a goal configuration. Many methods originating in robotics have exploited analogies between robot articulated chains and protein chains to model proteins. These methods either build over the Probabilistic Roadmap (PRM) planner [39,40] or tree-based planners, such as Rapidly-exploring Random Tree (RRT) [41], Expansive Spaces Tree (EST) [42], and Path-directed Subdivision Tree (PDST) [43]. In particular, PRM-based methods have been applied to fold small proteins [44,45]. Methods adapting tree-based planners have been proposed to model conformational changes and flexibility, predict the native structure, and even compute conformational paths connecting given structural states in proteins [46-51].

In particular, the T-RRT [50] and PDST-based method in [51] have addressed the problem of computing conformational changes connecting two given structures of a protein. While T-RRT has been shown to connect known stable structural states of the dialanine peptide (as the name indicates, the peptide is 2 amino acids long) [50], the PDST-based method has been shown to model the order of conformational changes connecting functional states of large proteins (200*-*500 amino acids long) [51]. In [51], the dimensionality of the conformational space is controlled through very coarse-grained representations essentially limiting the number of modeled parameters.

In this paper, we propose a novel robotics-inspired method to connect two given structural states of a protein by sampling conformational paths. The focus is on small- to medium-size proteins ranging from a few dozen to a few hundred amino acids (214 amino acids in the largest system). Rather than employing very coarse-grained representations to simplify conformational space, the proposed method models all backbone dihedral angles of a protein chain but relies on the molecular fragment replacement technique to efficiently model structural deformations. The technique essentially bundles together backbone dihedral angles and samples physically-realistic moves for them. The technique is popular in ab-initio structure prediction [47,52-54], but, to the best of our knowledge, has not been used before to model conformational changes.

The proposed method is tree-based. A tree, rooted at a given start structure, grows in conformational space in iterations. At each iteration, a conformation is selected for expansion. The expansion employs molecular fragment replacement technique and the Metropolis criterion to bias the tree towards low-energy conformations over time. Due to the employment of expansions and discretization layers to bias the growth of the tree, the method can be considered an adaptation of robotics EST and grid-based methods [40].

Like EST, the method pushes or expands the tree in the search space. It is worth noting that one reason we pursue an EST-based method is due to sampling and steering issues that have to be addressed in PRM- or RRT-based methods. In PRM, random configurations are first sampled in the configuration space, and a local planner is then used to connect neighboring configurations. Randomly sampled conformations have very low probability of being in a region of interest around intermediate states. In particular, for long chains with many degrees of freedom (hundreds of backbone angles in small-to-medium protein chains), a protein conformation sampled at random is very unlikely to be physically realistic. While biased sampling techniques can be used to remedy this issue [44,55], it is non-trivial to combine them with the molecular fragment replacement technique used here to reduce the dimensionality of the search space and obtain realistic protein- like conformations. In addition, both PRM and RRT rely on local planners or local deformation techniques to connect two neighboring conformations. In RRT, a conformation is sampled at random in conformational space, and a local planner attempts to connect it to its closest conformation in the tree. It is hard to find reasonable local planners for protein conformations. A linear interpolation is often carried over the degrees of freedom, typically backbone angles, but this can produce unrealistic intermediate conformations, and a lot of time can be spent energetically refining these conformations. Instead, the molecular fragment replacement technique can readily provide a physically-realistic child conformation when applied to a parent conformation. In effect, this allows addressing both the sampling and the steering issues.

For this reason, the method proposed in this paper pushes out in conformational space by expanding selected conformations in its search tree. A goal region is defined around the goal structure so that multiple paths can be obtained from one execution of the method. Since the objective is to steer the tree in one execution towards the given goal conformation, we experiment with different bias schemes both in the selection and expansion procedures. We refer to the bias over the selection as global bias, and that over the expansion as local bias. Local bias effectively allows making decisions on whether a generated child conformation should be added to the tree, only adding to the tree conformations that improve the proximity to the goal over the parent. Global bias allows steering the tree to meet various objectives. One objective is to reach the goal structure and so realize at least one path. Another conflicting objective is to prevent the tree from focusing only on certain regions of the conformational space and instead forcing it to maintain conformational diversity. Combined, meeting these two objectives allows balancing the exploration between progress to the goal and coverage of the conformational space so that diverse conformational paths are found and statistics can be computed over the transition ensemble.

A discretization layer is employed to implement a global bias towards the goal. The layer organizes sampled conformations in levels based on their proximity to the goal. A suitable progress coordinate, detailed below, is employed for this purpose. A weighting function can easily be defined over proximity levels, allowing to associate a probability distribution through which one can bias the selection of conformations for expansion to conformations close to the goal. We analyze different weighting functions, essentially implementing different bias strengths, for the trade-off between efficiency in reaching the goal versus premature convergence to local optima. Different bias strengths are also combined in a randomized strategy. A second discretization layer over the conformational space is considered here for its ability to prevent the tree from oversampling regions in conformational space. A low-dimensional embedding is associated with the conformational space, which allows defining a weighting function and so a probability distribution function used to bias the tree away from selecting conformations in overly-sampled regions of the conformational space. The incorporation of the second layer is shown to enhance path sampling by the method and even improve proximity to the goal. In addition, a reactive temperature scheme is investigated to further enhance sampling and allow paths to cross energy barriers as needed in a transition.

A preliminary proof-of-concept implementation of the tree-based exploration in the proposed method has been presented in [56]. Here we present a more general framework, where we additionally investigate the role of local bias schemes in the exploration, control the magnitude of jumps in conformational space in the expansion, employ an additional geometric projection layer to promote path diversity, and investigate a reactive temperature scheme to sample rare paths that cross high energy barriers. Additionally, a detailed investigation is conducted on the conformational paths modeled by the method on selected protein systems in comparison to available biophysical literature.

Our analysis here focuses on three systems that are well-characterized in literature, Trp-Cage, Calmodulin (CaM), and Adenylate Kinase (AdK). We show that the proposed method is effective in elucidating conformational paths on these systems. Moreover, due to the Metropolis criterion and a state-of-the-art energy function, the paths have credible energy profiles. The employment of a tolerance region around the goal structure allows obtaining many paths from one execution of the method. In the Results section, we employ multiple executions to obtain many paths, as commonly done by path sampling methods [50,51].

It is worth emphasizing that the paths computed by the method are not to be interpreted as transition trajectories. Instead, the conformations in them can be considered milestones during deformations of these paths into transition trajectories.

The proposed method makes an important contribution to the problem of modeling conformational changes employed by a protein to morph from a structure to another. On proteins where important functional states may be more than 13Å apart, it is not feasible to search for connecting conformational paths by sampling values for individual dihedral angles. On the other hand, as demonstrated by the work described in this paper, one does not have to resort to very coarse-grained representations to reduce the number of parameters. Instead, parameters can be bundled together and credible moves for them, extracted from known structures of proteins, can be proposed for a series of consecutive angles in order to efficiently obtain physically-realistic intermediate conformations. As we discuss in the Conclusions section, the method proposed here opens up new lines of investigation. The results in the Results section suggest that work in this direction is very promising to obtain credible conformational paths connecting diverse functional states of a protein. We now proceed with details on the proposed method.

## Methods

The tree-based exploration in the proposed method and interplay between selection and expansion has been previously presented in [56]. Our description of the proposed method focuses on the overall framework and novel components added to it in this paper. However, for completeness, we summarize the algorithmic components of the preliminary presentation in [56].

### Problem statement

The proposed method takes as input two structures (start and goal) corresponding to functional states. These structures can be obtained from the PDB. The method produces conformational paths. We define a path to be a series of conformations that terminates within some threshold distance of the given goal structure. The terms structure and conformation are often used interchangeably in computational structural biology literature. Structure refers to a specification of cartesian coordinates of atoms in a protein chain. Conformation refers to a spatial arrangement of the chain that can be internally represented through degrees of freedom other than cartesian coordinates. Indeed, in this paper we employ backbone dihedral angles, as detailed below. From now on, we will reserve the term structure to the input start and goal structures provided to the method. We will reserve the term conformation to those computed by the method and added to the tree in the search for conformational paths realizing the transition from the start to the goal structure.

As mentioned in the section "Experimental setup", a goal region is defined around the goal structure in order to obtain more than one path from one tree built during a single execution of the method. The goal region is defined through a threshold distance from the goal structure. We use least Root-Mean-Squared-Deviation (lRMSD) to measure distance from the goal structure. lRMSD is a semi-metric that measures structural dissimilarity as the weighted Euclidean distance between respective atoms in two aligned conformations (alignment finds the rigid-body transformation that minimizes RMSD, hence resulting in least RMSD) [57]. Low lRMSDs indicate high similarity. Interpretation remains difficult for lRMSDs > 6Å [58]. lRMSD is an imperfect measure, but simple to implement and employ as a progress coordinate when lacking any a priori information on reaction coordinates along which a transition of interest occurs.

We make use here the Metropolis criterion to limit the energetic difference between consecutive conformations in a path. The Metropolis criterion employs an effective temperature to control the height of energetic barriers that can be crossed by a path (detailed below). While most of the experiments in this paper are obtained with a medium temperature, a reactive temperature scheme is additionally investigated that adapts the probability for the conformational tree to cross energy barriers as needed. While a single execution of the method can provide multiple paths, more can be obtained from different executions. The paths can then be analyzed for their quality and diversity through clustering and other pseudo-free energy techniques to determine, for instance, highly-populated intermediates.

### Main algorithmic components of proposed robotics-inspired method

In the proposed method, a tree, rooted at a given start conformation, grows in conformational space in iterations. At each iteration, a conformation is selected in the tree for expansion. The expansion procedure produces conformations from a selected parent conformation, and a local bias scheme is investigated to determine whether a generated conformation should be added to the children of a parent in the tree. The selection procedure, which selects the conformation that should be extended at a given iteration, is key to balance different criteria, such as progress towards the goal and coverage of conformational space. The selection procedure employs one or more discretization layers and bias schemes over these layers to achieve one or both criteria. We refer to these as global bias schemes. We now relate details on the expansion and selection procedures.

#### Expansion procedure and local bias

The expansion procedure employs a coarse-grained representation, a coarse-grained energy function, and the molecular fragment replacement technique to generate candidate conformations from a selected parent conformation in the tree. We detail each of these components in order.

**Representation of a protein chain **The internal representation employed by the method is angular, and maintains only the *ϕ, ψ *backbone dihedral angles (there are 2*n *such angles for a chain of *n *amino acids). This is known as a kinematic model and allows drawing analogies with internal representations of robot articulated chains, where the only degrees of freedom used are revolute. Under this analogy, an atom corresponds to joint, and a bond connecting two atoms corresponds to a link. This angular representation for a protein chain is based on the idealized geometry assumption, which fixes bond lengths and angles between two consecutive bonds to idealized (native/equilibrium) values (taken from CHARMM22 [59] in this method) and limits variations to the backbone dihedral angles. The representation allows making use of the molecular fragment replacement technique described below to obtain a new conformation from a given one. After a conformation is obtained, forward kinematics is employed to compute cartesian coordinates for the modeled atoms from the backbone dihedral angles [60]. The only atoms modeled explicitly here are the *N *, *C*, *C_α_*, and *O *backbone heavy atoms. Side chains are sacrificed, as side-chain packing techniques can be used to add all-atom detail when necessary [61,62]. This angular representation and subset of modeled atoms are a common coarse-grained representation of protein chains, particularly in ab-initio structure prediction protocols for small- to medium-size proteins [63].

**Employed energy function **The function employed here to measure the potential energy of a generated conformation modifies the Associative Memory hamiltonian with Water (AMW) [64] studied extensively by us and others in modeling of native structures and equilibrium flexibility in proteins [48,49,53,54,65-68]. AMW is a linear combination of non-local terms (local terms are kept at ideal values in the idealized geometry model): *E*_AMW _= *E*_Lennard-Jones _+ *E*_H-Bond _+ *E*_contact _+ *E*_burial _+ *E*_water_. *E*_Lennard-Jones _implements the 12-6 Lennard-Jones potential as in AMBER9 [69], additionally allowing a soft penetration of van der Waals spheres. *E*_H-Bond _models hydrogen bonds. *E*_contact_, *E*_burial_, and *E*_water_, model formation of non-local contacts, a hydrophobic core, and water-mediated interactions. Further details can be found in [64,68].

**Molecular fragment replacement **The molecular fragment replacement technique is central to ab-initio structure prediction [70-73]. The key idea is that a subset of non-redundant structures are first obtained from the PDB. Configurations (*ϕ, ψ *angles) defined for *k *consecutive amino acids (fragments) are then excised from the PDB-obtained structures and stored in a fragment configuration library. Further details on the construction of the library are available in [49]. Here we use a fragment of length 3 rather than a fragment of more amino acids so as to limit the magnitude of the jump in conformational space resulting from one fragment configuration replacement (and thus the structural difference between two consecutive conformations in a conformational path sampled by the method). The fragment configuration replacement is conducted as follows. A position *i *in the chain is sampled uniformly at random, defining a fragment [*i, i *+ 2]. A fragment configuration (6 backbone dihedral angles) is then sampled uniformly at random over those available for the selected fragment in the library. The angle values of the sampled fragment configuration are then copied over those of the configuration of the selected fragment in the current conformation, resulting in a new conformation. This constitutes a move, accepted according to the Metropolis criterion detailed above. The key advantage over sampling individual dihedral angles is that the fragment configuration move is based on known structures, thus increasing the probability of obtaining a physically-realistic conformation.

**Putting it all together for expansion **The expansion procedure makes use of the molecular fragment replacement technique. A move sampled from a library of physically-realistic configurations, consisting of 6 backbone dihedral angle values, as described above, is proposed to modify the "parent" conformation selected for expansion to obtain a child conformation. The modification is accepted according to the Metropolis criterion with probability $e^{-\delta E/K{\;}_{B}\;T}$. *δE *is the energy difference between the resulting conformation and its parent, and *K_B _*is the Boltzmann constant. *T *is an effective temperature that serves as a scaling parameter through which the method controls the height of an energy barrier crossed by an acceptable child conformation. Most of our experiments detailed in the Results section employ a medium temperature, which allows the method to accept a 10 kcal/mol energy increase with probability 0.1. The Results section shows that this temperature is effective, but achieving connectivity in more complex systems, such as AdK, can benefit from the ability to cross higher-energy barriers. Adapting the temperature as needed by certain paths in the tree to cross energy barriers of varying heights is considered in this paper, and a reactive temperature scheme described below is implemented and analyzed in our experiments.

**Local bias in expansion procedure **We investigate a local bias in the context of the expansion to grow the tree with conformations that improve proximity to the goal. Essentially, moves are proposed until *m *conformations are obtained that all meet the Metropolis criterion. The maximum number of moves attempted is *l*. The conformation with the lowest lRMSD to the goal is considered for addition to the tree. Two different schemes are analyzed in this paper, one in which the child with the lowest lRMSD to the goal structure is added to the tree (this is the no local bias scheme), and another in which the addition is only carried out if the child's lRMSD to the goal is no higher than that of the parent (this is the local bias scheme). The local bias scheme essentially expands the tree only with a conformation that improves proximity to the goal over that of the parent. This is a greedy scheme that does not allow a path to veer away from the goal structure and possibly explore new transition routes. We compare both schemes in the Results section for how they affect the depth (progress towards the goal) and the breadth (path diversity) of the tree. While depth is measured as the lowest lRMSD to the goal structure over all paths that reach the goal region, a heuristic is introduced in the Results section to measure path diversity.

#### Selection procedure and global bias schemes over discretization layers

The selection procedure controls to a great extent where the tree grows in conformational space. Two discretization layers are considered for the selection procedure. While one is essential to the progress of the tree towards the goal, the other is considered to add conformational diversity and possibly obtain many uncorrelated paths from one execution of the method.

**Discretization layer over progress coordinate **Conformations in the tree are projected on a one-dimensional (1d) grid discretizing their lRMSDs to the goal. Grid boundaries are set at [0*, D*], where *D *is the lRMSD between the given start and goal structures. Conformations in the tree with lRMSD higher than *D *to the goal structure are mapped to the *D *level. Levels in the grid are separated by 1Å. The grid discretizes the explored conformational space in terms of the progress coordinate and allows biasing the growth of the tree towards the goal. While lRMSD is an imperfect measure, its employment as a progress coordinate has some precedent in biophysical studies that detect conformational transitions in CaM [21]. Using lRMSD as a progress coordinate, we investigate different bias schemes, as a strong bias towards selecting low-lRMSD conformations may perform well in a small system or in a particular run due to the probabilistic nature of the method and quickly drive the tree towards the goal. However, a strong bias may also lead to premature convergence to local optima and prevent the tree from approaching the goal. This is the classic depth vs. breadth issue that characterizes greedy exploration.

**Bias schemes over progress coordinate **Different bias schemes can be naturally defined through weighting functions over levels of the 1d grid. A quadratic function, referred to as QUAD, can be defined to associate a weight *w*(*l*) = 1*/*[1 + *l*^2^] + *∈*, with level *l *in the grid. The function biases the selection towards levels with low lRMSD to the goal, and *∈ *is set to a small value to ensure that higher-lRMSD conformations can be selected if the method is given enough time. Another weighting function, LINEAR, defined as *w*(*l*) = 1*/*[1 + *l*] + *∈*, reduces the bias. UNIFORM removes bias entirely, as in *w*(*l*) = 1*/*[#levels]. A probability distribution function then associates probability of selection $p\left(l\right)=w\left(l\right)/\left[\sum_{levelsl^{\prime }}w\left(l^{\prime }\right)\right]$ with a level *l*. Once a level *l *is selected with probability *p*(*l*), any conformation that maps to it is selected with equal probability for expansion. We also provide the first steps towards a probabilistic combination of different bias schemes. We compare the three basic bias schemes above to COMBINE_90-10_, which *p *= 90% of the time grows the tree with no selection bias (effectively employing UNIFORM), and 1 *- p *= 10% of the time employs QUAD. The value of *p *can be adaptively set in a reactive scheme to balance between tree depth and breadth, and this is a direction we will investigate in future work.

**Discretization layer over conformational space **A tolerance region of tolÅ around the goal structure allows defining a goal region and essentially obtaining many paths from one execution of the method. To some extent, the paths can be redundant, as the tree may waste time sampling similar conformations. An additional discretization layer can be defined over conformations in the tree, using shape- or contact-summarizing features (which we have previously studied for their ability to organize conformations [48,49,53]) to represent and project conformations. Here we investigate one such geometric projection of the conformations in the exploration tree. In addition to the 1d lRMSD grid described above, the 3 USR-based shape-summarizing features employed in [48] are used to associate a 3d grid with the tree. These features capture shape, as they are first momenta of atomic distance distributions in a conformation from selected points in the conformation. These points are chosen to be the centroid (ctd), the point farthest from the centroid (fct), and the point farthest from fct. These features essentially summarize a conformation with 3 coordinates and thus allow projecting conformations in the tree over a 3d grid.

**Bias scheme over geometric projection layer **A second weighting function can be defined now over this 3d grid to bias the tree away from similar conformations. The weighting function we use here is 1.0*/*[(1.0 + **nsel**) * **nconfs**], where **nsel **records how often a cell is selected, and **nconfs **records the number of conformations that project to the cell. This function essentially penalizes with higher weights (and thus, lower probability of selection) cells of the 3d grid that have been selected many times before and/or have already contain many conformations in them. This function has been used by us in previous studies on enhancing conformational sampling for ab-initio protein structure prediction. Our investigation of this additional discretization layer, presented in the Results section shows that this forces the tree to find diverse paths without adversely affecting the ability of the tree to reach the goal structure.

**Putting it all together for selection **When employing both discretization layers, the selection scheme would first select a level over the 1d grid over the progress coordinate with probability of selection dependent on the weighting function used. Many conformations in the tree would correspond to the selected lRMSD level. Rather than selecting any conformation in that level uniformly at random (which is the case if the second discretization layer is not used), all cells in the 3d grid where these conformations map would be available if the second discretization layer is used. The weighting function over the 3d grid allows associating probability of selection to these cells. After a cell is selected, any conformation in it can be selected uniformly at random to be a parent for the expansion procedure.

### Controlling magnitude of jumps in conformational space for sufficient path resolution

The purpose of the discretization layers and the bias schemes detailed above is to possibly obtain diverse conformational paths that reach the defined goal region. There are no additional constraints that these path have sufficient resolution in them. There is nothing to prevent a path reaching the goal region with one or just a few conformations. To some extent, this is a consequence of the granularity of the moves employed to generate conformations. The fragment replacement technique can make larger jumps in conformational space than if single dihedrals were sampled at each iteration of the tree. However, the bundling of dihedral angles together is necessary to be able to traverse the space in a reasonable amount of time. Providing some path resolution, where possible, is appealing. Greater conformational detail along a possible transition trajectory alleviates the task for techniques that will spend their time on pursuing deformations of these paths into actual transition trajectories.

We pursue the following simple scheme to control the magnitude of the jump in conformational space in the expansion procedure where possible. From all the candidates that pass the Metropolis criterion and local bias conditions (if applicable), we calculate the mean and standard deviation of the lRMSDs of candidate conformations to the parent conformation. We then sample a value from a Gaussian distribution defined with these parameters and select the candidate conformation whose lRMSD to the parent is closest to the sampled value.

### Reactive temperature scheme

Reactive schemes that change the temperature as needed to make progress, introduced in [50] for the dialanine peptide system, present an interesting direction to further enhance the exploration of the method we propose here. Building on this body of work, we investigate here a simple reactive scheme that responds to global measurements made on the conformational tree at regular intervals during the execution of the method. The progress towards the goal structure is monitored over every *w *iterations with no overlap (the tree grows by one conformation in each iteration), effectively sliding a window of length *w *over the fixed number of iterations for which the method is run. If the lowest lRMSD to the goal structure by any of the conformations added to the tree during those *w *iterations in window *i *is not less than some value *d*_1 _than the lowest lRMSD over window *i *- 1, then the temperature is increased. If the lowest lRMSD achieved over window *i *is at least *d*_2 _lower than the lowest lRMSD achieved over window *i *- 1, the temperature is decreased.

The motivation for monitoring the tree over every *w *iterations is that a global decision can be made on possibly many paths not able to improve the overall progress of the tree to the goal structure. When improvements are not made, this is indicative that many paths are not able to add conformations that meet both the Metropolis criterion and extend the tree towards the goal. This means that there are energetic barriers that the current temperature does not allow crossing, and this necessitates a temperature increase. While temperature increases enhance the exploration capability, they also do not allow sufficiently sampling a local minimum by effectively increasing the magnitude of jumps that the tree makes in conformational space with every added branch. Therefore, the balance between exploration and exploitation is restored by lowering the temperature when improvements in lRMSD exceed a threshold. While large improvements may seem desirable, it is worth noting that the purpose for the method is not to quickly reach the goal structure with possibly few very long branches. Instead, the goal is to produce a series of conformations that capture the transition in some detail. Therefore, lowering the temperature effective limits the magnitude of the jumps that the tree can make in conformational space with each branch and so provide some level of resolution in the transition from the start to the goal structure.

**Figure 1:**
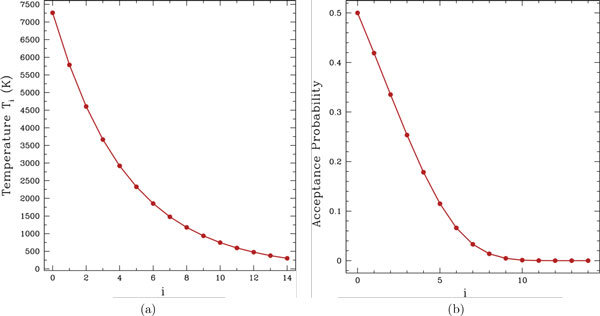
**(a) Proportional cooling scheme used for the reactive temperature setting is shown**. Temperatures go down from *T*_0 _to *T*_14_. (b) The corresponding acceptance probabilities, under the Metropolis criterion, are shown, using *δE *= 10 kcal/mol.

The temperatures considered are obtained from a proportional cooling scheme often used in the context of simulated annealing. They go from a high temperature *T*_0 _*≈ *7261K down to room temperature *T*_14_=300K over 15 cooling steps. The fixed medium temperature used for a part of our experiments that do not employ the reactive temperature scheme corresponds to *T*_9. _These temperatures define acceptance probabilities, under the Metropolis criterion. *T*_0_ is defined so that the acceptance probability under it is 0.5 for an energetic increase of 10kcal/mol. *T*_0 _is lowered 15 times according to a proportional cooling schedule that updates the temperature as in *T_i_*_+1 _= *T_i _*(*T*_14_*/T*_0_)*^k^*_+1_) until *k *= 14. The temperatures and their corresponding acceptance probabilities for an energetic increase of 10 kcal/mol are shown in Figure 1. This proportional cooling scheme has been employed by us before for simulated annealing in [68]. The reactive temperature scheme employed in this paper starts with *T*_9_. The scheme then iterates over the temperatures. If the current temperature employed by the method is *T_i_*, where 0 ≤ *i *≤ 14, and the reactive scheme demands lowering it, then the temperature is set to *T_i_*_+1_. If the scheme demands increasing it, then the temperature is set to *T_i-_*_1_. The lowest temperature allowed is *T*_14_, and the highest allowed is *T*_0_.

## Results

### Implementation details and experimental

We select here for our analysis three well-characterized systems, Trp-Cage, CaM, and AdK. These systems' respective lengths are 20, 144, and 214 amino acids (aa). The method is executed 10 times on each system. Each execution terminates when adding to the tree 1, 000 conformations for Trp-Cage and 10, 000 conformations for CaM and AdK (since Trp-Cage is a small system, our investigation shows that 1, 000-2, 000 conformations yield similar overall performance). One execution of the method on one CPU takes 1-2 minutes on Trp-Cage, 8 hours on CaM, and 24 hours on AdK. Multi-threaded executions cut down time requirements by a factor of 10. About 90% of CPU time is spend on energy function evaluations.

The tolerance around the goal structure to define the goal region is dependent on protein size. On Trp- Cage, tol = 1Å. On CaM and AdK, tol is set to 4 and 5Å, respectively. The maximum number of moves attempted in the selection procedure is *l*=100, and *m*=10 candidates are generated from a selected parent that all satisfy the Metropolis criterion. The window size *w *used to monitor the progress of the tree in terms of lowest lRMSD in the reactive temperature scheme is set to 100 iterations. There is no window overlap. The value of the *d*_1 _parameter defining minimum required improvement is set to 0.25Å. The value of the *d*_2 _parameter is set to 1.5Å.

### Experimental setup

The performance of the method is summarized in terms of depth versus breadth. Depth is defined as the lowest lRMSD reached by the tree to the goal structure. An estimate of breadth over paths that reach the goal region is defined as $b=\left(\sum_{i=o}^{h}\left(i+1\right)\cdot d_{i}\right)/h$, where *h *is the number of nodes on the shortest path, and *d_i_* is the maximum pairwise lRMSD among conformations atfA second weighting function can be defined now over this 3d grid level *i *across all paths (*i *grows from goal to root). This measure downweights differences in lower levels (closer to the goal).

A total of five settings are considered: (i) only one discretization layer is used in the selection procedure, and four different bias schemes are considered over the progress coordinate. No local bias is employed in the expansion procedure; (ii) local bias is added in the expansion procedures; (iii) the magnitude of the junp in conformational space in the expansion procedure is restricted through the step size mechanism described in Methods; (iv) A second discretization layer is added over a geometric projection of the conformational space; (v) A reactive temperature scheme is considered as opposed to a fixed-temperature exploration.

We apply the method on the following systems and transitions. On Trp-Cage, we connect an extended conformation to the native structure (PDB id 1l2y). On CaM, we analyze the ability to connect all 6 directed pairs that can be defined over its three functional states. These states are documented under PDB ids 1cfd (apo), 1cll (holo), and 2f3y (collapsed). CaM is an ideal system to study, as it is a key signaling protein in many cellular processes exhibiting a particularly large conformational rearrangement. On AdK, a variety of states have been reported, but we focus here on the most studied transition from the apo/open (PDB id 4ake) to the closed state (PDB id 1ake).

### Comparison of global bias schemes over progress coordinate

A summary of the method's performance in terms of depth (we recall that depth is the lowest lRMSD obtained by the method to the given goal conformation) is shown in Table 1. We focus here on the first setting, where no local bias is implemented in the expansion procedure. All four bias schemes on the progress coordinate are tested in the selection procedure. Results are averaged over 10 independent executions of the method, and Table 1 shows averages (*μ*) and standard deviations (*σ*) in depth across the different global bias schemes considered. The results obtained under QUAD, LINEAR, UNIFORM, and COMBINE_90-10 _are reported in columns 3-6, respectively.

**Table 1:** Average (*μ*) and standard deviations (*σ*) are reported for the lowest tree lRMSD over 10 executions of the method.

System	Start *→ *Goal	*μ ± σ *over lowest lRMSDs (Å)			
		QUAD	LINEAR	UNIFORM	COMBINE_90-10_
Trp-Cage (20 aa)	E *→ *1l2y (18 Å)	1.91 *± *0.53	2.32 *± *0.78	2.34 *± *0.59	2.42 *± *0.43

	1cfd *→ *1cll (10.7 Å) 1cll *→ *1cfd (10.7 Å)	3.22 *± *0.13 3.42 *± *0.24	3.49 *± *0.42 3.66 *± *0.33	3.69 *± *0.26 3.97 *± *0.17	3.36 *± *0.13 3.49 *± *0.24
	
CaM (140 aa)	1cfd *→ *2f3y (9.9 Å) 2f3y *→ *1cfd (9.9 Å)	3.83 *± *0.43 3.50 *± *0.26	3.76 *± *0.52 3.54 *± *0.37	4.23 *± *0.31 3.80 *± *0.17	4.01 *± *0.34 3.57 *± *0.28
	
	1cll → 2f3y (13.44 Å) 2f3y → 1cll (13.44 Å)	1.76 ± 0.53 0.86 ± 0.25	1.52 ± 0.31 0.80 ± 0.20	1.44 ± 0.25 1.06 ± 0.31	1.50 ± 0.20 0.94 ± 0.14

AdK (214 aa)	1ake → 4ake (6.95 Å) 4ake → 1ake (6.95 Å)	4.20 ± 0.51 4.48 ± 0.86	4.39 ± 0.47 5.62 ± 0.80	5.47 ± 0.28 5.94 ± 0.15	4.32 ± 0.41 5.09 ± 0.69

Weighting schemes for global bias over node selection are compared here. No local bias is used in the expansion procedure.

Table 1 shows that the method is able to reach the goal. On Trp-Cage, the average lowest lRMSDs are 1.9-2.4Å, which is comparable with the 1.5-2.5Å range reported by folding and structure prediction studies [48,74]. The test on Trp-cage, which is a small system, is intended to show the basic capability of the method. The paths are not to be interpreted as folding paths, as the fragment configurations are extracted from native/folded protein structures. On CaM, the average lowest lRMSDs obtained by the method range from sub-angstrom to slightly over 4Å, depending on the particular pair of start and goal structures connected. The results show that some pairs are more difficult to connect than others. On the 1cfd to 1cll paths, the average lowest lRMSDs are below 4Å, which is in general agreement with the 1.5-5Å proximity reported by MD-and MC-based studies [21,75]. AdK represents a more challenging case for method. Lowest lRMSDs obtained here are 4-6Å, slightly higher than the 2.5Å obtained with coarse-grained models by the PDST-based method in [51].

**Figure 2:**
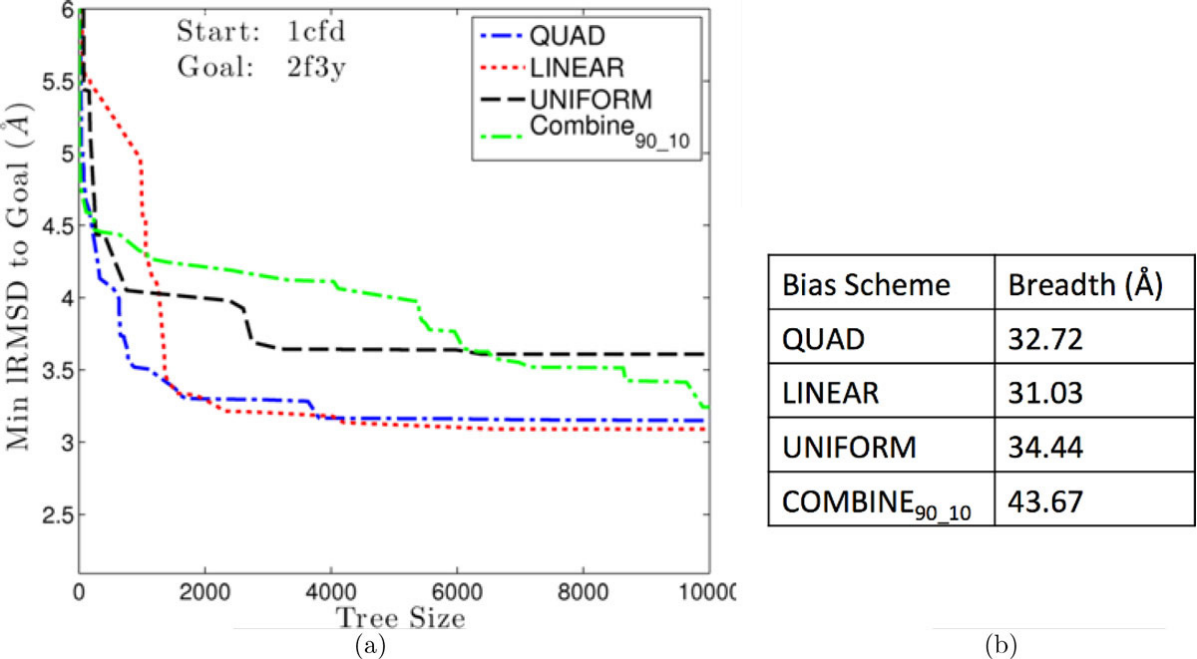
**(a) Minimum lRMSDs to the goal structure are plotted as a function of tree size and compared among global bias schemes**. No local bias is employed in the expansion procedure. (b) Global bias schemes are additionally compared in terms of path diversity.

The results shown in Table 1 make the case that all global bias schemes allow the method to reach the goal. Here we take a closer look at how these schemes do so over the time. We focus on one of the transitions in CaM and the "best" execution (over 10) that allows a bias scheme to achieve its lowest lRMSD to the goal structure. Figure 2(a) highlights the expected behavior, showing that while QUAD can drive the exploration rapidly towards the goal, it may plateau for long periods of time. LINEAR shows a similar rate of descent, followed by COMBINE_90-10_. Of all bias schemes, UNIFORM and COMBINE_90-10 _exhibit a more gradual decrease in lRMSD, suggesting that the exploration is more diverse under these two schemes. We recall that, while the tree is not globally biased towards the goal under UNIFORM, a conformation added to the tree in the expansion procedure is chosen to be the one closest to the goal among energetically-credible conformations generated from a selected conformation (this is the 'no local bias' setting). In Figure 2(b) we analyze path breadth or diversity on the same 1cfd to 2f3y transition. Figure 2(b) shows the breadth estimate across all bias schemes and confirms that diversity is higher in UNIFORM and COMBINE_90-10_. Taken together, these results suggest that the COMBINE_90-10 _global bias provides the right compromise between depth and breadth.

#### Comparison of schemes in expansion procedure

The experiments detailed above are repeated to measure the effect of adding a local bias in the expansion procedure (which only adds the candidate with lowest-lRMSD to the goal structure as the child of a parent node if its lRMSD to the goal is also less than that of the parent conformation to the goal) and controlling the magnitude of the conformational jumps from parent to child (described in Methods as limiting step size). In order not to add too many constraints for the expansion procedure, the step size is not controlled when incorporating local bias in the expansion procedure.

**Figure 3:**
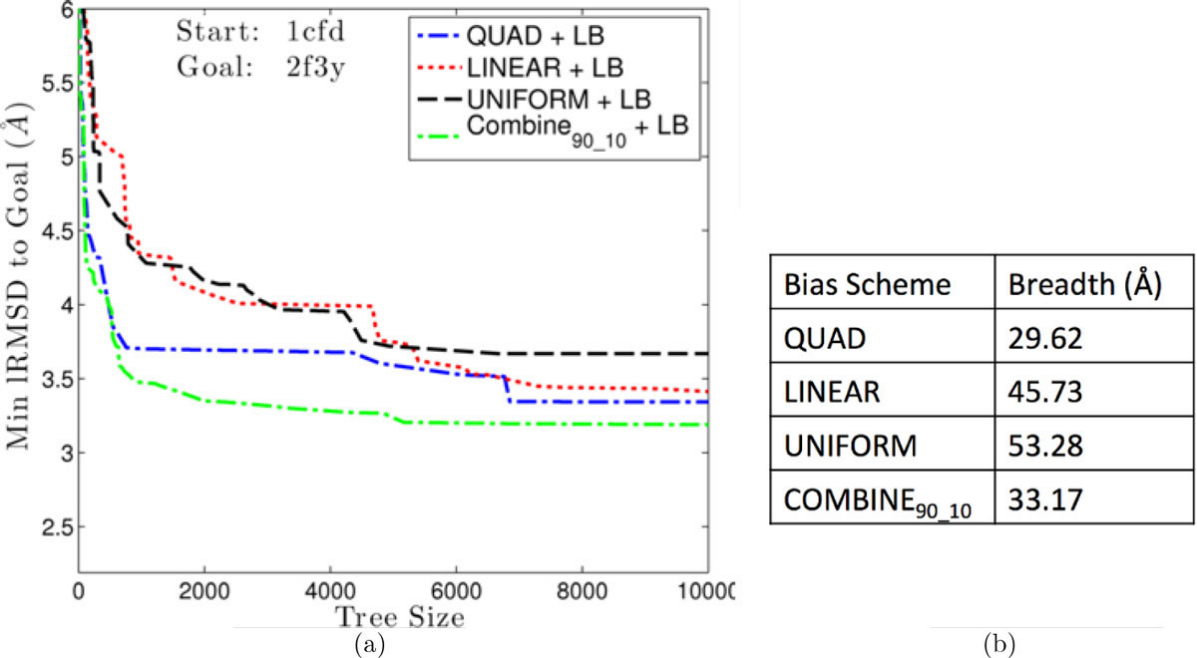
**(a) Minimum lRMSDs to goal are plotted as a function of tree size and compared among bias schemes**. Local bias is employed in the expansion procedure.(b) Global bias schemes are additionally compared in terms of path diversity.

Detailed results obtained when incorporating local bias in the expansion procedure are reported in columns 3-6 in Table 2. Overall, introduction of the local bias does not significantly improve the ability of the method to get closer to the native structure, but lower lRMSDs to the goal are obtained over the baseline setting when no local bias is implemented in the expansion procedure. For instance, on Trp-Cage, the average lowest lRMSDs are now 1.8-2.2, which is a slight improvement over the baseline setting of no local bias. On the 1cfd to 2f3 transition in CaM and vice versa, the average lowest lRMSDs are now consistently under 4Å. Slight improvements are also obtained on the AdK closed-to-open transition and vice versa. As above, an additional analysis shown in Figure 3(a) tracks minimum lRMSD to the goal over the tree during the execution of the method and compares breadth among the different global bias schemes. Similar to the results shown above for the baseline setting of no local bias, QUAD plateaus early. The decrease in lowest lRMSD to the goal is more gradual under UNIFORM and LINEAR. The best improvement is obtained by COMBINE_90-10_. The comparison of breadth values in Figure 3(b) shows that LINEAR and UNIFORM have the highest breadth, followed by COMBINE_90-10_. Taken together, these results suggest that adding local bias in the expansion procedure does not significantly improve proximity to the goal structure, but it may limit diversity. In both settings, COMBINE_90-10 _provides a compromise between depth and breadth.

**Table 2:** Average (*μ*) and standard deviations (*σ*) are reported for the lowest tree lRMSD over 10 executions of the method.

System	Start *→ *Goal	*μ ± σ *over lowest lRMSDs (Å)			
		QUAD+LB	LINEAR+LB	UNIFORM+LB	COMBINE_90-10_+LB
Trp-Cage (20 aa)	E *→ *1l2y (18 Å)	1.86 *± *0.53	2.12 *± *0.57	1.83 *± *0.63	1.87 *± *0.43

CaM (140 aa)	1cfd *→ *1cll (10.7 Å) 1cll *→ *1cfd (10.7 Å)	3.17 *± *0.25 3.35 *± *0.51	3.27 *± *0.10 3.56 *± *0.29	3.49 *± *0.26 3.70 *± *0.23	3.32 *± *0.12 3.50 *± *0.21
	
	1cfd *→ *2f3y (9.9 Å) 2f3y *→ *1cfd (9.9 Å)	3.93 *± *0.37 3.43 *± *0.39	3.93 *± *0.42 3.65 *± *0.45	3.99 *± *0.24 3.62 *± *0.13	3.76 *± *0.41 3.34 *± *0.13
	
	1cll → 2f3y (13.44 Å) 2f3y → 1cll (13.44 Å)	1.91 ± 0.58 0.82 ± 0.30	1.67 ± 0.49 0.72 ± 0.08	2.01 ± 0.86 1.02 ± 0.43	1.68 ± 0.37 0.73 ± 0.10

AdK (214 aa)	1ake → 4ake (6.95 Å) 4ake → 1ake (6.95 Å)	3.91 ± 0.34 4.65 ± 0.71	4.28 ± 0.36 5.32 ± 0.79	5.15 ± 0.30 5.62 ± 0.37	4.19 ± 0.22 5.21 ± 0.41

Weighting schemes for global bias over node selection are compared here. Local bias is incorporated in the expansion procedure.

**Figure 4:**
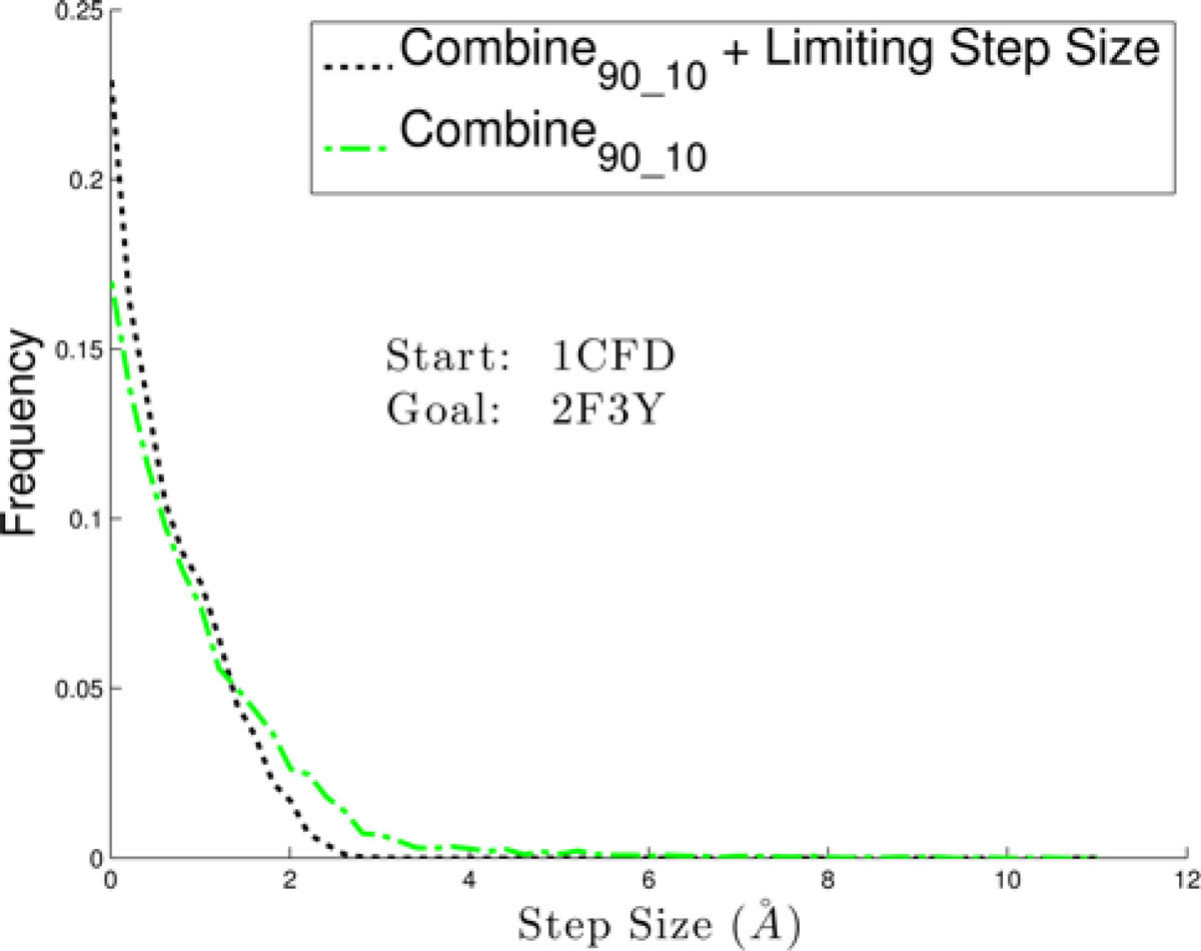
**Step size is controlled in the expansion procedure**. Step size is measured as the lRMSD between a parent and child in a tree. The distribution of step sizes in the exploration tree is highlighted on one selected transition for CaM, over all global bias schemes when using no local bias in the expansion procedure.

Rather than adding local bias in the expansion procedure, one can try to limit the magnitude of a move from parent to child in the tree in order to provide some minimal path resolution. We now do so by limiting the size (lRMSD) of a branch from parent to child (step) as described in the Methods section. Figure 4 compares the distribution of step sizes in the exploration tree as a result of limiting them with the procedure described in the Methods section to the underlying distribution in the baseline setting where step sizes are not controlled. The comparison focuses on the 1cfd to 2f3y transition in CaM, employing COMBINE_90-10 _for the global bias over the progress coordinate). Figure 4 allows drawing two conclusions. First, the Metropolis criterion in the expansion procedure implicitly biases step sizes even when no additional control is applied over them. Most step sizes are no more than 2Å. Second, explicitly controlling the step size as described in the Methods procedure is effective and does not significantly change the underlying distribution significantly. The procedure described to control step sizes aims to center them around 2Å, which is not very hard to do, as seen in the underlying distribution.

**Figure 5:**
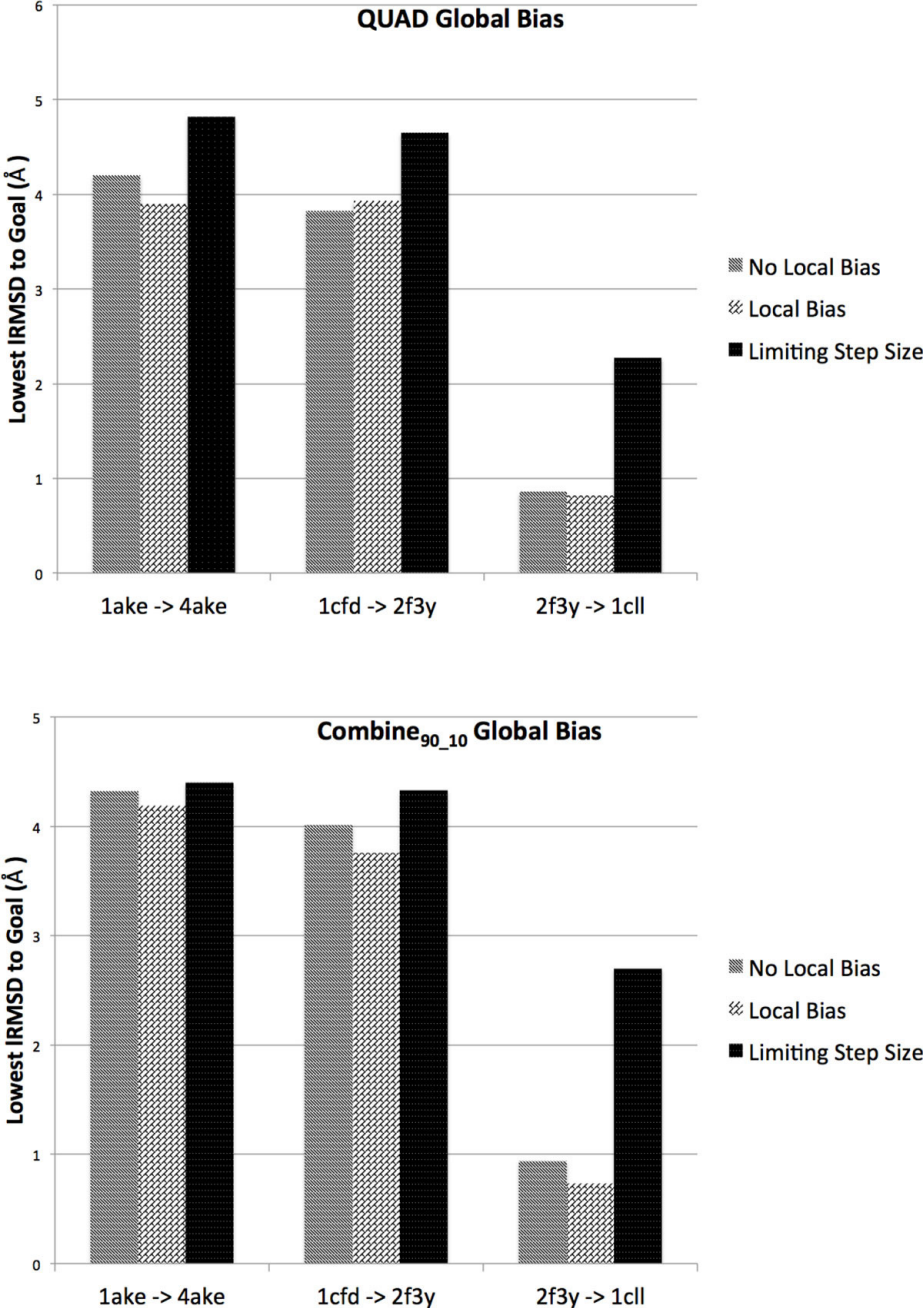
**Depth is compared across the three different local schemes considered in the expansion procedure**. The global bias schemes considered are QUAD (top) and COMBINE_90-10 _(bottom). The comparison is highlighted on three selected transitions.

We now analyze the effect that explicit control over step sizes has on the ability to reach the goal. Figure 5 shows the depth reached when controlling the step size on three selected transitions of medium- to high- difficulty for the method (as determined on the baseline setting of no local bias in the expansion procedure). The best run over 10 is shown. The depths reached on each of the three selected transitions are visually compared to those obtained when not controlling the step size, whether incorporating local bias or not in the expansion procedure. Again, the best run is shown for these other settings in terms of depth. The global bias schemes considered here are either QUAD or COMBINE_90-10_. Figure 5 shows that, when limiting the step size, it is harder for the method to achieve similar proximity to the goal structure. Most decreases in proximity to the goal are less than 1Å. A higher decrease of about 2Å is observed for the 2f3y to 1cll transition in CaM.

The adverse effect on proximity when limiting the step size is expected. Demanding more resolution along conformational paths in the tree distributes more conformations and computational resources to obtaining more intermediate points along a path rather than extending paths toward the goal structure. Increasing the size of the conformational tree allows obtaining similar depth when controlling the step size to the other two settings. This is observed when running the method to sample 25, 000 rather than 10, 000 conformations (data not shown).

### Analysis over incorporating geometric discretization layers

**Figure 6:**
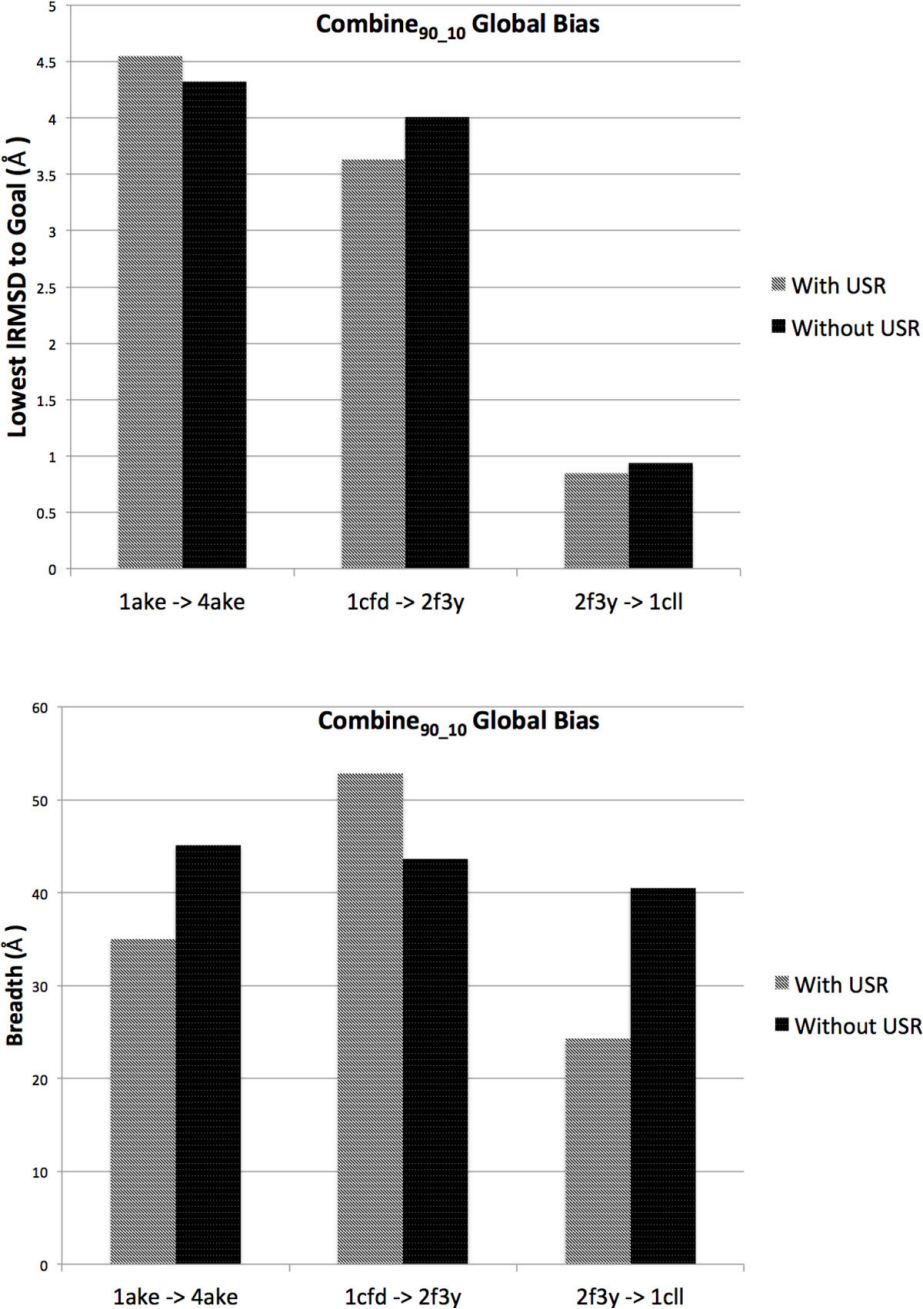
**Depth (left) and breadth (right) are compared when using the second discretization layer ('with USR' in legend) over not using it ('without USR')**. The 'without USR' setting is the baseline setting where no local bias is employed in the expansion procedure. The global bias scheme considered here is COMBINE_90-10_. The comparison is highlighted on the same three selected transitions.

In this setting we add the second USR-based discretization layer, thus discouraging the tree from visiting the same regions in conformational space too often. As described in the Methods section, this is achieved by projecting conformations in the tree onto a 3d grid. We limit the analysis here to the setting of using the COMBINE_90-10 _global bias scheme over the progress coordinate for the first discretization layer and employing no local bias in the expansion procedure. Figure 6 compares the depth (top row) and breadth (bottom row) of the tree obtained when incorporating the geometric projection layer as opposed to not incorporating it. The shown values for depth and breadth correspond to the run that achieves the best depth (lowest lRMSD to the goal) over 10 runs.

The comparison shows that insisting on diversity does not significantly hamper the tree from reaching the goal structure with similar lowest lRMSDs. Differences in depth are within 0.5Å. In fact, on two transitions, slight improvements are obtained. It is important to note that the extent of improvements of depth depends both on the extent of sampling and on whether paths need to be fine tuned or altogether alternative routes have to be found. When fine tuning is needed, a finer granularity in the 3d grid for the geometric projection of the conformational space may help further improve proximity to the goal. Comparison of breadth values shows that the improvements in breadth and depth are correlated. This is a consequence of the fact that, when the projection layer increases lRMSD to the goal, fewer paths are considered successful and counted in the breadth analysis.

### Analysis over incorporating reactive temperature scheme

All of the above experiments employ a fixed temperature corresponding to *T*_9 _in the temperature schedule shown in the Methods section. Here we consider a reactive temperature scheme, as described in the Methods section, to enhance sampling and allow paths to jump over energy barriers as needed. In this setting, we set the maximum number of moves attempted in the expansion procedure to *l*=250, and *m*=25 candidates are generated from a selected parent that all satisfy the Metropolis criterion. When increasing the temperature, the exploration is more likely to yield conformations farther in conformational space, and so it is harder to obtain children that approach the goal. The higher number of moves and children allow us to address this.

**Figure 7:**
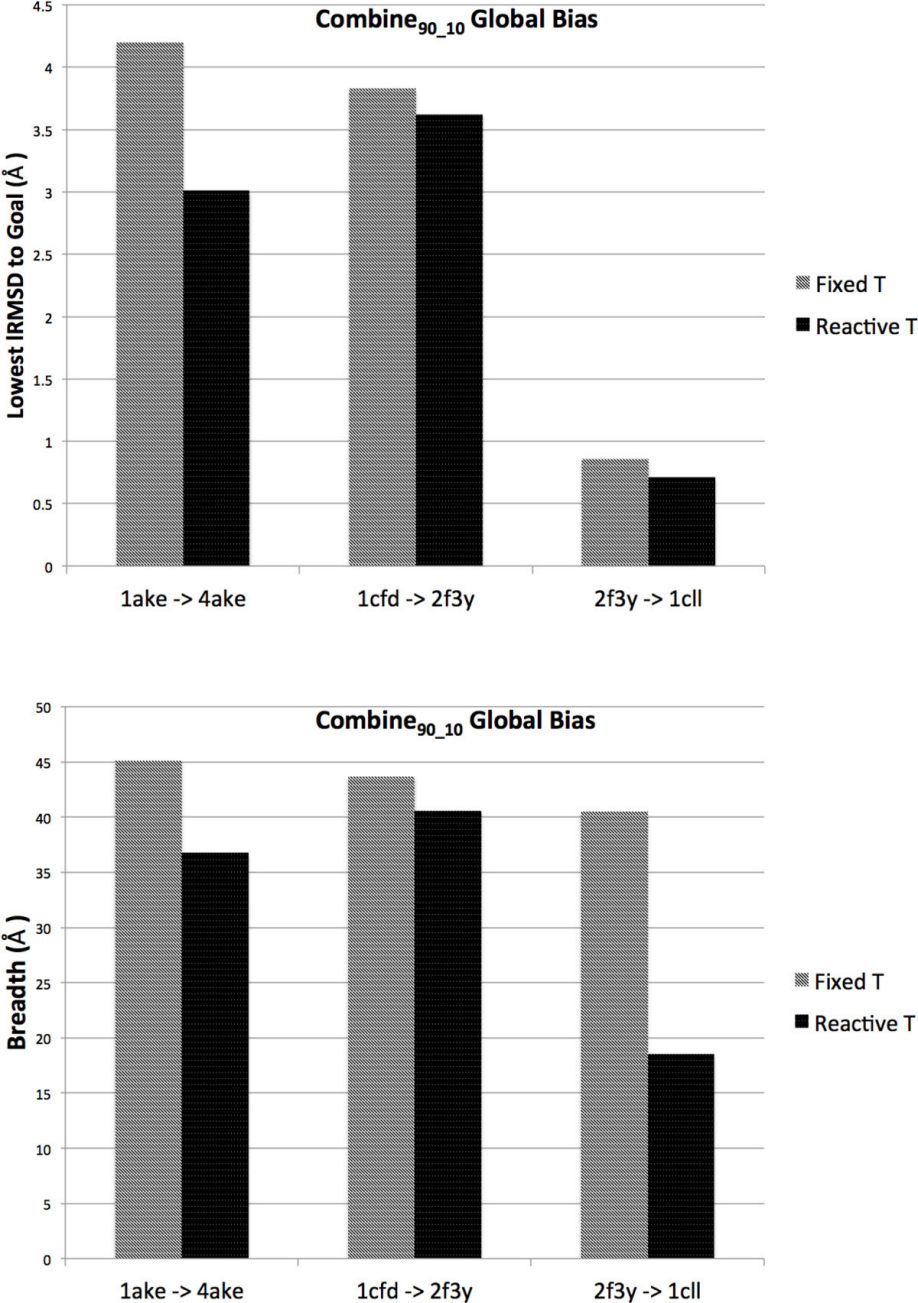
**Depth (left) and breadth (right) are compared when using the reactive temperature scheme ('reactive T' in legend) as opposed to using a fixed temperature ('fixed T')**. The 'fixed T' setting is the baseline setting where no local bias is employed in the expansion procedure. The global bias scheme considered here is COMBINE_90-10_. The comparison is highlighted on the same three selected transitions.

Figure 7 shows the depth reached when incorporating the reactive temperature scheme on the same three selected transitions of medium- to high-difficulty for the method. The best run over 10 is shown. The global bias scheme employed over the progress coordinate is **COMBINE**_90-10 _(only one discretization layer is used), and no local bias is used in the expansion procedure. The depths reached on each of the three transitions are visually compared to those obtained when employing a fixed temperature (*T*_9_) instead of the reactive scheme. Figure 7 shows that the reactive temperature improves depth for all three transitions. Further analysis of depth shows that the reactive temperature scheme provides the best improvement, by more than 1Å in the case of AdK. This transition is difficult, and the improvement in depth by allowing paths to cross energy barriers suggests that the transition goes over high-energy regions. In the other two transitions, where the baseline setting of the method achieves good proximities to the goal structure, the reactive temperature scheme offers slight improvements in proximity to the goal. Breadth is also higher, which suggests that more paths are sampled by the method when allowed to jump energy barriers.

### Detailed analysis on CaM transition ensemble

As shown above, the method is able to surpass lRMSDs of >13.44Å on CaM, even obtaining sub-angstrom lRMSDs to the goal when setup to approach 1cll from 2f3y (slightly higher 1-2Å lRMSDs are obtained in the other direction). The above analysis seems to suggest that connecting the other 4 directed pairs is more difficult; lowest lRMSDs across all bias schemes are in the 3-4Å range. The additional employment of the USR-based discretization layer offers slight improvements in these difficult cases.

**Figure 8:**
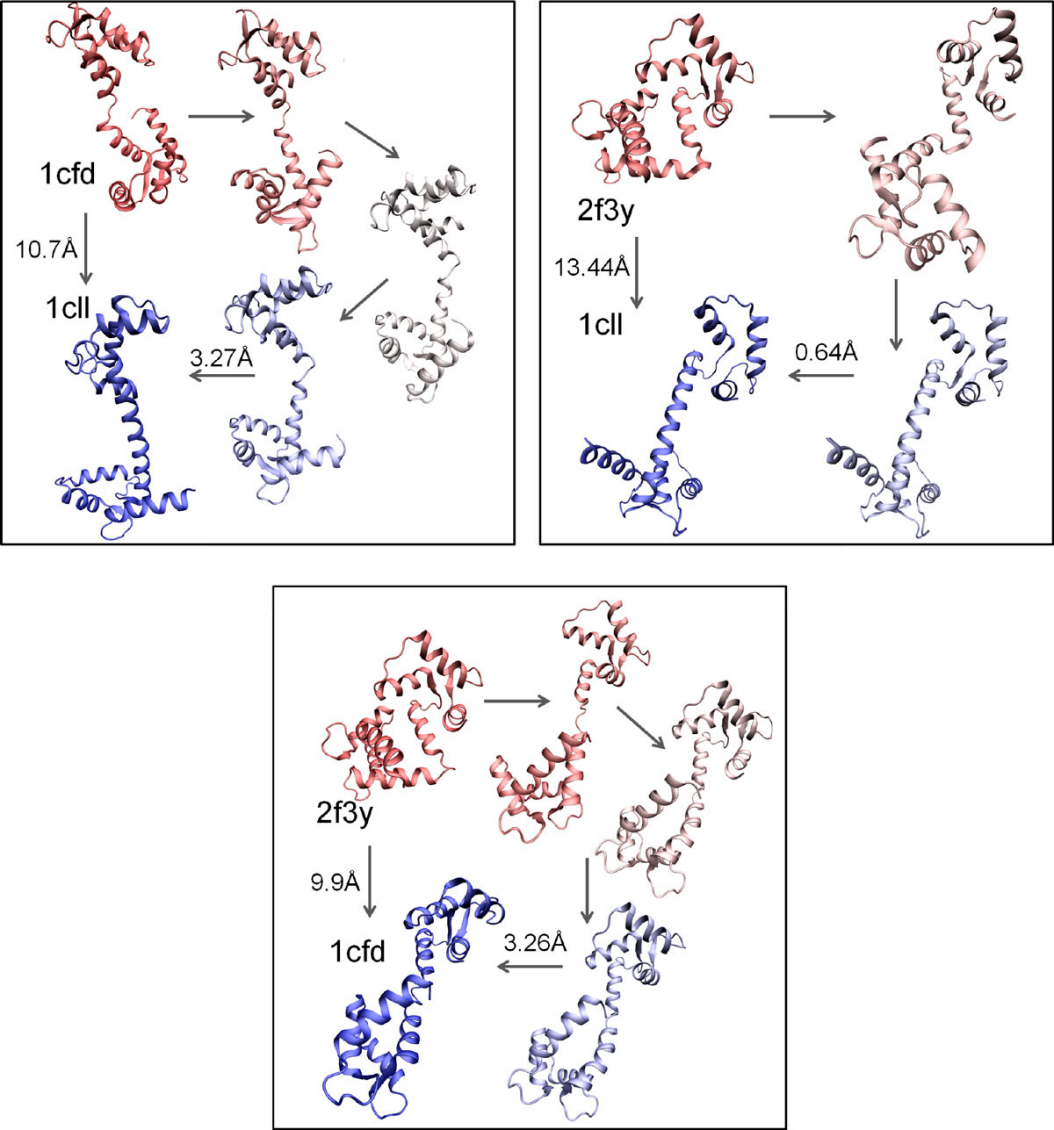
**Three paths are highlighted**. Start and goal structures are in red and blue, respectively. Selected conformations in the path are drawn in a red-to-blue interpolated scheme.

The results on CaM agree qualitatively with those observed in experiment and simulation studies [21,76,77]. The transition between 1cll and 2f3y is easier than between the other pairs. Though the other pairs have initial lRMSDs that are lower, the true distance that has to be surpassed is in angular space, which partially explains why, by employing the molecular fragment replacement technique, the method performs well. We highlight some paths showing these angular rearrangements in Figure 8.

**Figure 9:**
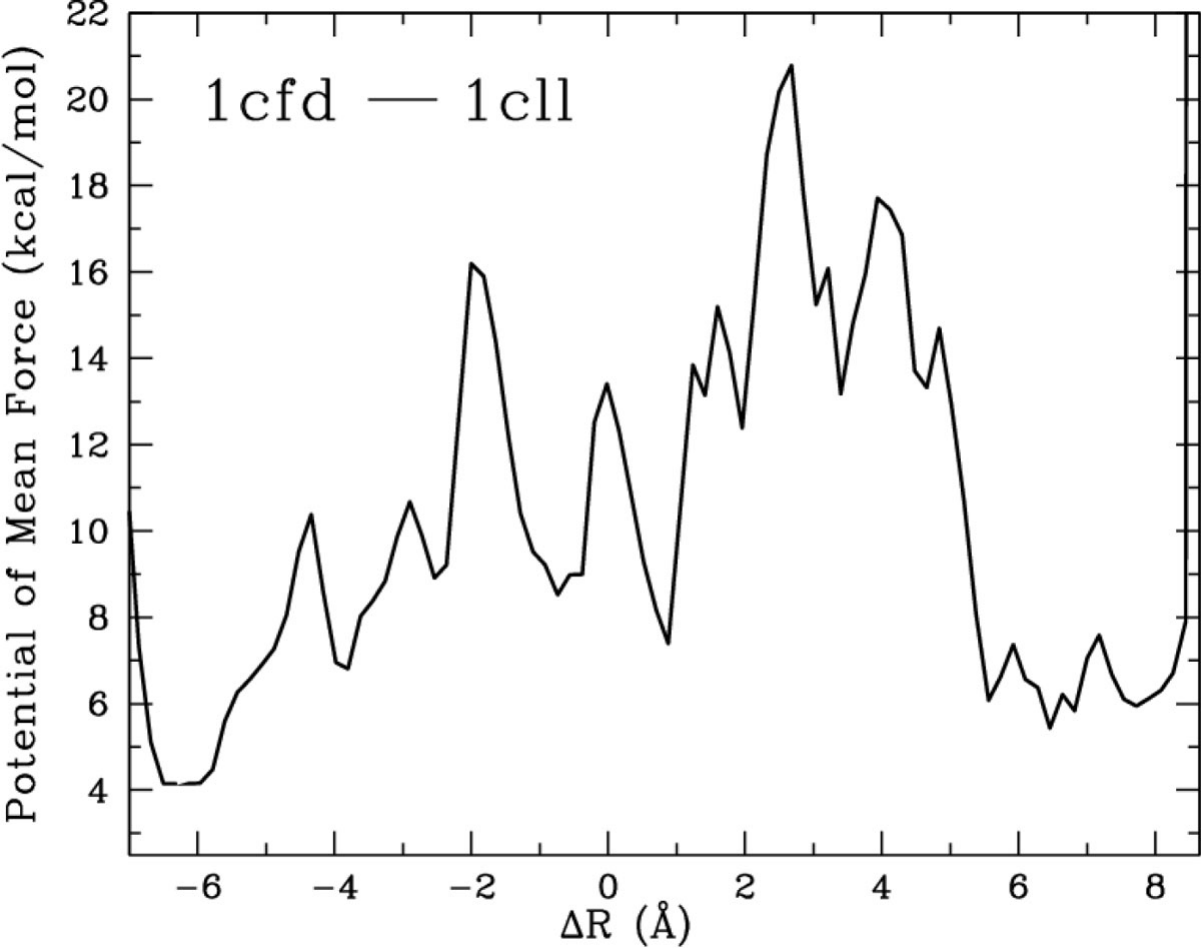
**Pseudo-free energies along Δ*R *are shown for sampled paths connecting 1cfd to 1cll and vice versa**.

It is worth noting that the use of fragments is justified when transitions do not involve unfolding, which is the case on many proteins, including CaM and AdK. Wet-lab studies on CaM exclude the possibility that functional transitions involve a significant population of unfolded or disordered states [77]. These studies also suggest that the transition between 1cfd and 1cll is complex and surpasses energy barriers. To illustrate this, a pseudo-free energy landscape is associated with the conformations sampled by our method and shown in Figure 9. Paths from 10 executions of the method are obtained, using COMBINE_90-10 _and local bias in the expansion procedure on connecting 1cfd to 1cll and vice-versa. Pseudo-free energies are calculated along the Δ*R *coordinate (defined as lRMSD(*C, C*_1cfd_) - lRMSD(*C, C*_1cll_)) employing the weighted histogram analysis method (WHAM) [78]. The pseudo-free energy landscape in Figure 9 shows that paths have to cross regions of high free energy, qualitatively agreeing with wet-lab findings in [77]. The shown pseudo-free energies need to be taken with caution, as they are affected by potential low sampling density and path diversity (we discuss these issues in section 2).

### Detailed analysis on AdK transition ensemble

**Figure 10:**
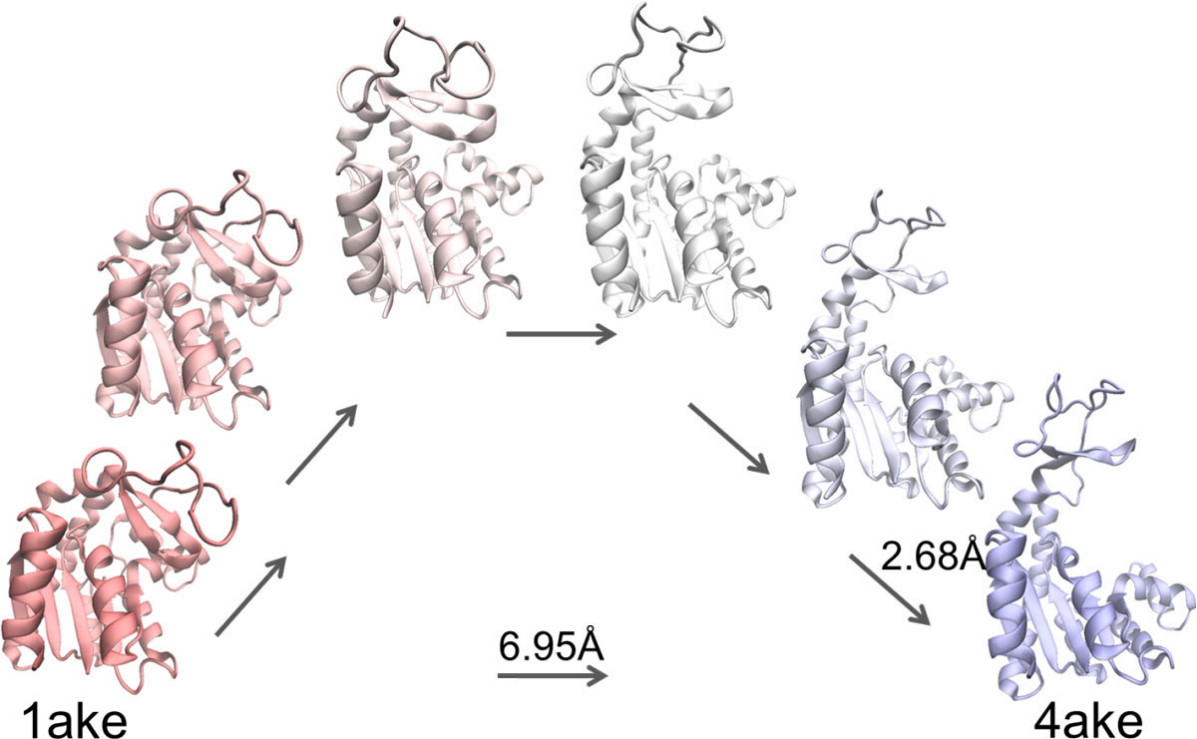
**A path capturing the transition from 1ake to 4ake is shown here**. Start and goal structures are in red and blue, respectively. Selected conformations in the path are drawn in a red-to-blue interpolated scheme.

The transition from the closed (corresponding to PDB id 1ake) to the open state (PDB id 4ake) in AdK has been the subject of many recent studies. We show in Figure 10 a sample path capturing the conformational change from the closed to the open structure. This path, which reaches the goal structure with an lRMSD of less than 3Å, is the best one in terms of depth obtained with the reactive temperature scheme, using COMBINE_90-10 _as the global bias scheme over the progress coordinate, and using no local bias in the expansion procedure. This path shows the opening of the two domains in the structural transition.

Studies on modeling the closed to open transition reproduce the presence of many known intermediate structures [10,51,79]. In particular, in [10], all known crystal structures are analyzed for their presence in the closed to open transition. Here we conduct a similar analysis after collecting all intermediate structures deposited for AdK in the PDB. Some of these structures have been captured on systems with slight sequence variations (due to the experimental technique extracting them from different species). As in [10], we employ the SwissView homology-modeling server [80] to thread these structures onto the amino-acid sequence of 1ake so that a direct analysis can be performed in terms of lRMSD.

We measure the extent to which we find each of the 27 crystal structures as intermediate conformations (in terms of lowest lRMSD) over all paths that reach the goal within 3.5Å. We limit the analysis to the above setting of employing the COMBINE90_10 global bias scheme, using no local bias for the expansion procedure, and incorporating the reactive temperature scheme. We report the minimum lowest lRMSD over the best path over all runs (best in terms of depth). Table 3 reports two minimum lowest lRMSDs per structure, one for the 1ake to 4ake transition and the other for the 4ake to 1ake transition. The PDB ids of the crystal structures are shown in column 1. The ordering is indicative of the location of these structures in the 1ake to 4ake transition (structures listed at the top are closer to 1ake, and those at the bottom are closer to 4ake). Some of the known intermediate structures are in dimeric configurations in the crystal (chains A and B are available in the PDB), but we employ here only chain A for analysis, since the chains are structurally identical. Table 3 shows that the paths in the 1ake to 4ake transition capture most of the known intermediate structures with lowest lRMSDs below 3Å, which suggests that the method captures well the presence of known intermediates and is able to model the 1ake to 4ake transitions in AdK. On the reverse transition, the higher lRMSDs indicate that there are possibly high local maxima that limit the exploration capability and the quality of paths.

**Table 3:** The lowest lRMSD to each of the known crystal structures for AdK is calculated over all paths that reach the goal within 3.5Å.

PDB id	lowest lRMSD (Å)	
	1ake*→*4ake	4ake*→*1ake
1e4v	0.32	3.17
1e4y	0.93	3.02
2eck	0.35	3.17
1ank	0.48	3.13
1zin	2.24	2.76
1zio	3.75	3.19
1zip	2.09	2.79
1s3g	1.71	3.31
1p3j	1.48	3.37
2eu8	1.31	3.04
2p3s	1.47	3.30
2oo7	1.26	3.06
2ori	1.30	3.04
2osb	1.31	3.07
2rh5	3.82	2.82
2rgx	2.93	3.34
1aky	1.44	3.12
2aky	1.30	3.30
3aky	1.41	3.14
1dvr	2.91	3.02
1zak	2.81	3.99
2ar7	3.65	3.10
2bbw	3.79	3.38
2c9y	3.17	3.73
1ak2	2.94	4.04
2ak2	2.89	3.97
2ak3	3.78	3.73

The value shown in column 2 is the minimum lowest lRMSD obtained over the best run (in terms of depth) of the method using the COMBINE_90−10 _global bias scheme over the progress coordinate, no local bias for the expansion procedure, and the reactive temperature scheme for the 1ake to 4ake transition. Column 3 shows the minimum lowest lRMSD for the 4ake to 1ake transition. Column 1 shows the PDB id of each of the crystal structures considered. The structures are ordered according to their locations along the 1ake to 4ake transition.

This preliminary study on AdK is promising However, AdK presents an extremely challenging case for our method, not only due to its size but also due to the presence of a significant energy barrier in the transition [13]. Tables 1 and 2 show that lowest lRMSDs can be above 4Å. Lack of density in sampling makes a pseudo-free energy analysis premature. In addition to more sampling, complex proteins, such as AdK, may present additional challenges in silico due to possibly more complex energy surface. The above analysis of the effect of the reactive temperature, however, shows that proximity to the goal structure can be improved when the temperature is changed by the method as needed for paths to cross energy barriers. This suggests that the energy landscape of AdK is much more complex, with transition states of potentially high energies.

## Conclusions

This paper proposes a robotics-inspired tree-based method to compute conformational paths connecting functionally-relevant states of a protein. The method makes use of the molecular fragment replacement technique to efficiently grow the tree with physically-realistic conformations. Consequently, paths sampled by the method are largely applicable to connecting states that do not involve unfolding. External factors in conformational switching (e.g., presence of binding partners) are not considered. Instead, the conformational selection model is employed, under which diverse functional states co-exist at equilibrium albeit with different probabilities even in the absence of interacting molecular partners for the protein of interest [81,82].

The approach proposed in this paper is a first step in elucidating the series of conformations that span a structural transition between two stable states of a protein molecule. We believe this is the first employment of an EST-based method in combination with the molecular fragment replacement technique and representational coarse graining. The detailed analysis in this paper shows that the method computes credible conformational paths connecting given structural states in small- to medium-size proteins. Various settings in terms of global and local bias schemes are investigated in this paper for how they affect the ability of the method to reach the goal structure while retaining path diversity. Soft global bias schemes over the chosen progress coordinate, lRMSD to the goal, and no local bias schemes in the expansion of the tree are found to be effective. Addition of a second discretization layer that biases the tree away from visiting similar regions in conformational space is found to improve proximity to the goal when increasing path diversity.

Controlling the step size to provide more resolution along paths can hamper the ability to reach the goal structure with low lRMSDs when the size of the tree is limited to 10, 000 conformations. Larger trees are found to recover the proximity even when the step size is limited. Controlling the step size, to the extent that the moves employed by the algorithm allow doing so, is important. Since these conformational pathways will be ultimately the subject of deformation techniques that include dynamics, better resolution is expected to provide better guidance for the deformation. We note that adding path resolution can be performed as a post-processing step, in which case it can be limited only to paths that reach the goal structure.

A reactive temperature scheme is implemented in this paper that allows the method to jump energy barriers of varying heights. The temperature is increased if progress towards the goal structure slows done, and is brought back down if progress fastens. A proportional temperature schedule is employed. The reactive temperature scheme is shown to considerably improve the ability of the method to reach the goal structure in the difficult transition considered here for AdK. This suggests that on proteins where the energy surfaces are complex and transitions go over high-energy barriers, a reactive temperature scheme can be effective. These findings support other studies where alternatively-implemented reactive temperature schemes allow connecting stable states of small peptides [50,83].

Several directions of research can be further pursued. For instance, MD-based techniques can be employed to deform paths into actual transition trajectories and thus obtain credible timescale information. Additionally, path smoothing techniques can be investigated. Reactive schemes can also be implemented over the global bias schemes to allow the method to switch between different global bias schemes as needed to adaptively balance the exploration between breadth and depth. Progress coordinates other than lRMSD to the goal can also be investigated. We have already investigated a coordinate based on contact topology and structural profiles [53]. However, this coordinate is shown to be useful only when differences in contacts between the goal and start structures are significant (data not shown). On most functionally-relevant structures, differences are limited to only a few contacts.

It is worth noting that sampling multiple structurally-diverse conformational paths with statistical rigor is an important issue that needs to be addressed in future work. We propose that, due to incorporation of discretization layers, the method described in this paper provides a natural bed on which to investigate balancing progress and coverage. Obtaining a comprehensive view of paths connecting two structural states of a protein allows identification of possibly interesting intermediate stable and semi-stable states and additional rigorous calculation of barriers in free energy landscape for direct quantitative validation with experimental and computational biophysical studies of structural transitions in proteins.

## Competing interests

The authors declare that they have no competing interests.

## Authors' contributions

KM suggested the methods and the performance study in this manuscript and drafted the manuscript. AS guided the study, provided comments and suggestions on the methods and performance evaluation, and improved the manuscript writing.

## Declarations

The publication costs for this article were funded by the corresponding author AS through NSF Grant Nos. 1016995 and 1144106.

This article has been published as part of *BMC Structural Biology *Volume 13 Supplement 1, 2013: Selected articles from the Computational Structural Bioinformatics Workshop 2012. The full contents of the supplement are available online at http://www.biomedcentral.com/bmcstructbiol/supplements/13/S1.
